# From Sudoscan to bedside: theory, modalities, and application of electrochemical skin conductance in medical diagnostics

**DOI:** 10.3389/fnana.2024.1454095

**Published:** 2024-10-23

**Authors:** Benjamin Vittrant, Hanna Ayoub, Philippe Brunswick

**Affiliations:** Withings, Issy-les-Moulineaux, France

**Keywords:** electrochemical skin conductance (ESC), SUDOSCAN, Withings, review, opinion

## Abstract

The human body has two main types of sweat glands: apocrine and eccrine. Eccrine glands are widely distributed across the skin, including areas with hair. While the eccrine glands on palms and soles help improve grip, those on the rest of the body primarily aid in thermoregulation. Sudomotor function, which controls sweating, is regulated by the sympathetic division of the autonomic nervous system through cholinergic and adrenergic pathways. The activation of eccrine glands involves intricate processes, including neurotransmitter binding, ion channel modulation, and voltage generation. Sudoscan technology utilizes electrochemical skin conductance (ESC) to non-invasively measure sudomotor function. This method, which has been standardized for accuracy, has established normative benchmarks and has proven reliable across diverse populations. Sudoscan’s diagnostic performance is comparable to invasive methods such as intraepidermal nerve fiber density testing, making it a valuable tool for diagnosing small fiber neuropathy. Moreover, it has been shown to correlate with corneal nerve fiber length, providing insights into various neuropathic conditions. Compared to traditional sudomotor function tests, Sudoscan proves superior in terms of its accessibility, simplicity, and reliability, with the potential to replace or complement existing diagnostic methods. It is important to differentiate ESC, as measured by Sudoscan, from other skin conductance measures, such as galvanic skin response (GSR) or electrodermal activity (EDA). Although these methods share a common physiological principle, ESC is specifically designed for diagnosing sudomotor function, unlike GSR/EDA, which is typically used for continuous monitoring. Sudoscan’s success has led to its integration into consumer health devices, such as the BodyScan from Withings, showcasing its versatility beyond clinical settings. Future research may explore ESC applications in diverse medical fields, leveraging real-world data from integrated consumer devices. Collaborative efforts between researchers and engineers promise to offer new insights into sudomotor function and its implications for broader health monitoring. This study provides a comprehensive overview of ESC, including topics such as eccrine gland physiology, sudomotor function, Sudoscan technology, normative benchmarks, diagnostic comparisons, and potential future applications.

## Introduction

Peripheral neuropathies (PNs) (Saporta et al., 2023; Hanewinckel et al., 2016) encompass a range of medical conditions caused by damage to the peripheral nervous system, which serves as an extensive communication network that transmits information between the central nervous system (the brain and the spinal cord) and the rest of the body. This complex system is responsible for relaying various types of sensory information, such as the sensation of cold feet, as well as transmitting commands from the brain to the body, including those that control voluntary muscle movements and vital involuntary functions such as heart rhythm, blood flow, digestion, and immune response.

PNs can be divided into mononeuropathies (Mononeuropathy, 2023), multifocal neuropathies (Neuropathy, 2023), and polyneuropathies (Polyneuropathy, 2023) and can be classified based on the type of nerve fibers involved: motor, sensory, or autonomic nerves.

Neuropathy most commonly takes the form of polyneuropathy, where many or most nerves throughout the body are affected. Symptoms of peripheral neuropathy vary widely and can range from mild to debilitating, though they are seldom life-threatening. Symptoms can develop gradually or suddenly and may improve spontaneously without extensive treatment. Unlike the nerve cells in the central nervous system, peripheral nerve cells have the ability to regenerate throughout life. Some of the more common PNs include diabetic neuropathy (Tesfaye et al., 2010), Guillain-Barré syndrome (Habib and Waheed, 2023), carpal tunnel syndrome (Malakootian et al., 2023), meralgia paresthetica (Scholz et al., 2023), and complex regional pain syndrome (Shafiee et al., 2023; Sobeeh et al., 2023). Treatment and management of peripheral neuropathy depend on the underlying cause and the severity of the symptoms. A multidisciplinary approach, including medication, physical therapy, lifestyle modifications, and, in some cases, surgery, can help manage symptoms and improve the quality of life for those affected by this condition.

PN diseases affecting the peripheral autonomic nerves are called peripheral autonomic neuropathies (PANs). The autonomic nervous system (Gibbons, 2019) regulates almost all involuntary body functions, such as heart rate, digestion, and sweating. It is divided into three parts: the sympathetic, parasympathetic, and enteric systems (Intestine Innervation, 2023). Damage to the autonomic nerve can cause symptoms such as excessive sweating, heat intolerance, blood pressure irregularities, gastrointestinal issues, and, in rare cases, difficulty swallowing when the esophageal nerves are involved (Kaur et al., 2021; Golden and Vernino, 2019; Roth et al., 2021; Lamotte and Sandroni, 2022).

The current gold standard for assessing small peripheral nerve fibers is measuring epidermal nerve fiber density (ENFD) via a skin biopsy (Basantsova et al., 2019). A nerve biopsy involves surgically removing and microscopically examining nerve tissues, typically from a sensory nerve in the lower leg, such as the sural nerve (Nolano et al., 2020; Lauria et al., 2010). While this procedure provides detailed information regarding the specific types of nerve cells and subcellular structures that are involved in the disease process, it is important to note that a nerve biopsy is an invasive procedure that carries the risk of causing additional nerve damage, potentially resulting in persistent neuropathic pain and sensory deficits in the affected area.

To address these risks, neurodiagnostic skin biopsies (Nolano et al., 2020; Buonocore, 2014; Sommer, 2018) were developed as a less invasive alternative. This procedure involves removing a small skin sample, usually approximately 3 mm in diameter, under local anesthesia. This method allows healthcare professionals to evaluate the nerve fiber endings within the skin. Skin biopsies have become the preferred method for diagnosing small fiber neuropathies, particularly those undetectable by traditional nerve conduction studies or electromyography (EMG). Although more sensitive than symptom-based assessments and reflex testing (Hlubocky et al., 2010), both skin and nerve biopsies remain invasive, costly, and sometimes inconsistent.

Recognizing these limitations, Philip Low (Morgan, 2018) at the Mayo Clinic in Rochester (Minnesota, United States) developed a method to assess PANs. He introduced a novel approach for evaluating sweat production to quantify sudomotor function, which led to the creation of the Quantitative Sudomotor Axon Reflex Test (QSART) (Riedel et al., 1999; Thaisetthawatkul et al., 2013). By employing pharmacological agents to stimulate sweat glands, Low demonstrated the clinical relevance of sudomotor testing in diagnosing autonomic neuropathies across various conditions, including diabetes. Despite its utility, the pharmacological stimulation used in QSART presents challenges in terms of quantification and reproducibility (Berger and Kimpinski, 2013; Buchmann et al., 2019).

Additionally, accurately measuring the small volumes of sweat produced (in microliters) necessitates a controlled environment with precise temperature and humidity regulation. QSART assesses sweat gland function at multiple body sites, including the forearm, proximal and distal leg, and foot. However, the test focuses on a limited number of glands within these regions and thus depends on the region. It is also important to note that QSART specifically targets the cholinergic nerves, which are responsible for stimulating sweat secretion, and does not assess other types of nerve fibers. These drawbacks have limited its widespread clinical use.

However, the groundwork laid by Low led to the development of a new approach by Impeto Medical, based on electrochemical skin conductance (ESC). ESC uses low direct and step currents to activate the sweat glands, functioning as a stress test to evaluate the small nerve fibers innervating these glands. This approach resulted in FDA clearance of a device, the Sudoscan^@™^ (K100233 & K141872) (U.S. Food and Drug Administration, 2023a,b), and various clinical advancements, with a historical focus on diabetic neuropathies (Casellini et al., 2013; Gatev et al., 2020; Oh et al., 2022; Selvarajah et al., 2015).

Given its high reproducibility (Casellini et al., 2013; Calvet et al., 2016; Riveline et al., 2023), ESC has been adopted in numerous fields, and independent recent reviews have extended its application to PANs in general (Lefaucheur et al., 2018; Lefaucheur, 2023). Interest in the technology is reflected in Pubmed search results: a query for “Sudoscan” yielded 161 publications from 2011 to September 20, 2024, with 14, 21, 23, 22, and 19 entries for the years 2023, 2022, 2021, 2020, and 2019, respectively, indicating growing interest and usage of the technology.

In 2023, the technology was integrated into a consumer scale (Body Scan^@™^) (Riveline et al., 2023) from Withings Inc. (2008) manufacturer. This integration was a giant step for the ESC since it allowed the study of time profiles for patients while allowing the building of an enormous data set that is available for medical research. Current research is ongoing to understand the doors opened by this integration, which allows patients to take measures at home and favors remote patient monitoring (RPM).

This work explores the biology and physics behind electrochemical skin conductance (ESC), while providing a refresher on the basics of sudomotor function. We will also clarify how ESC differs from sympathetic skin response (SSR), also known as electrodermal activity (EDA). Although both techniques rely on similar biological mechanisms, we will explain why they should not be used interchangeably. Our goal is to make this review accessible to a non-technical audience and present the advantages and limitations of ESC as clearly as possible, enabling physicians and researchers to consider its use in their practice. The main corpus is structured as follows:

- Underlying biology of the sudomotor function
- Electrochemical skin conductance: historical concept and technical development
- Electrochemical skin conductance: validation and clinical development
- Discussion, Conclusion, Limitations

## Sudomotor function

The human body contains two primary types of sweat glands: apocrine and eccrine. While all mammals possess eccrine sweat glands (ESG) on their footpads—referred to as palms and soles in primates and humans—humans are distinct in having millions of ESG spread across the entirety of their skin, including areas with hair. Eccrine glands are solitary structures that are uniformly distributed across the skin surface, although their concentration varies depending on the location, with the highest densities found on the palms and soles. Despite their structural similarities and the production of comparable aqueous sweat, eccrine glands are categorized into two separate groups based on their distinct functions and attributes. Those located on the palms and soles serve a specialized purpose: they produce sweat that enhances friction and improves grip. On the other hand, eccrine glands across the rest of the body are primarily involved in thermoregulation, helping to maintain a stable body temperature. Both types of eccrine glands are regulated by the hypothalamus and are predominantly activated by cholinergic stimuli, which are also involved in processes such as salivation and digestion (Cui and Schlessinger, 2015; Marples, 1965).

Reflecting their divergent evolutionary paths, the eccrine glands on the palms/soles and those on the rest of the body develop at different stages in human fetal growth, with the former appearing around the 16th week and the latter around the 22nd week of gestation (Cui and Schlessinger, 2015; Montagna, 1984; Cui et al., 2014; Tafari et al., 1997).

**Figure 1:**
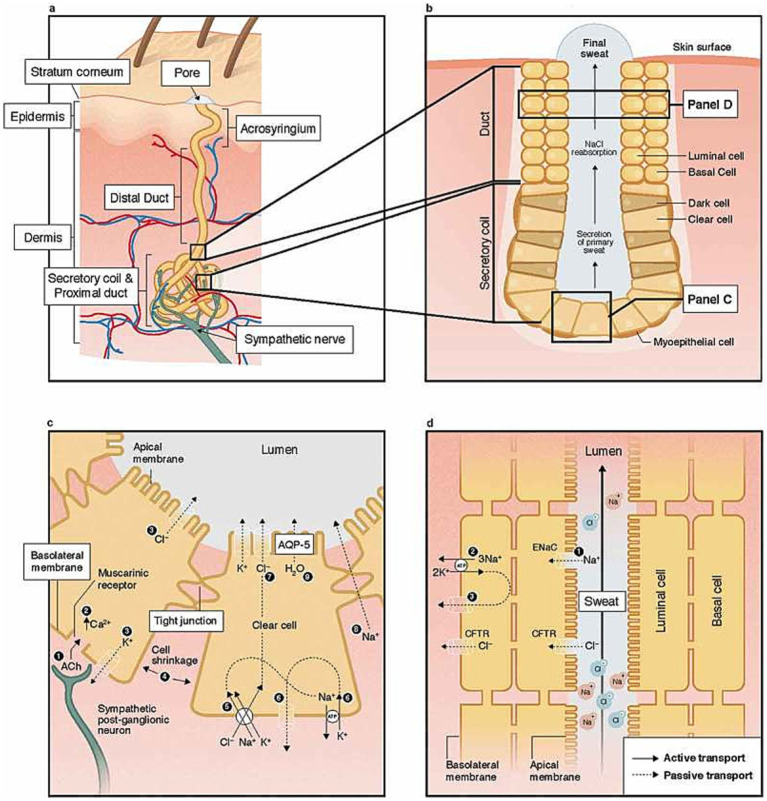
Structure of the human eccrine sweat gland at skin level **(a)**, pore level **(b)**, cell levels **(c)**, and **(d)**. Reprinted from Baker (2019), licensed under CC BY-NC-ND 4.0.

A good exhaustive and detailed description of sweat gland biology can be found in 2 papers from L. B. Baker: *Physiology of Sweat Gland Function: The Roles of Sweating and Sweat Composition in Human Health* (Baker, 2019) and *Physiological Mechanisms Determining Eccrine Sweat Composition* (Baker and Wolfe, 2020). Our following and summarized description came from those two papers while our Figure 1 is directly taken from the first paper (their figure is under Creative Commons Attribution-NonCommercial-NoDerivatives License^1^.)

ESG operates independently of the hair follicles and sebaceous (oil-producing) units (Baker, 2019; Baker and Wolfe, 2020). They consist of a coiled tubular epithelium, which, in the secretory part of the coil, is accompanied by myoepithelial cells (Figure 1a,b). These cells contract to facilitate the expulsion of sweat. Sweat is predominantly composed of sodium (Na+) and chloride (Cl−) and is responsible for polarization, but other compounds like lactate potassium can be found at various levels. Details about sweat composition can be found in Table 1 from *Physiological Mechanisms Determining Eccrine Sweat Composition* (Baker and Wolfe, 2020). Within the deep dermal layer of the skin, the coiled part of the gland is intimately associated with nerve endings (Figures 1a,c), small pockets of fat, and blood vessels, which together contribute to the gland’s function and regulation (Sonner et al., 2015).

Recent research also showed the role of the ESG in wound healing (Rittié et al., 2013), making it a target for healing process follow-up. By controlling the skin PH and dryness, the ESG can drive to or away from specific physico-chemical conditions to modulate the bacterial population. In the case of DPN, sweat dysfunction disturbs this balance to allow bacterial growth and invasion that can lead to open wounds and diabetic foot ulcer (DFU) in the ultimate stage (Rittié et al., 2013; Martin, 1997; Diao et al., 2019; Burgess et al., 2021; Evans and Sen, 2023).

**Figure 2:**
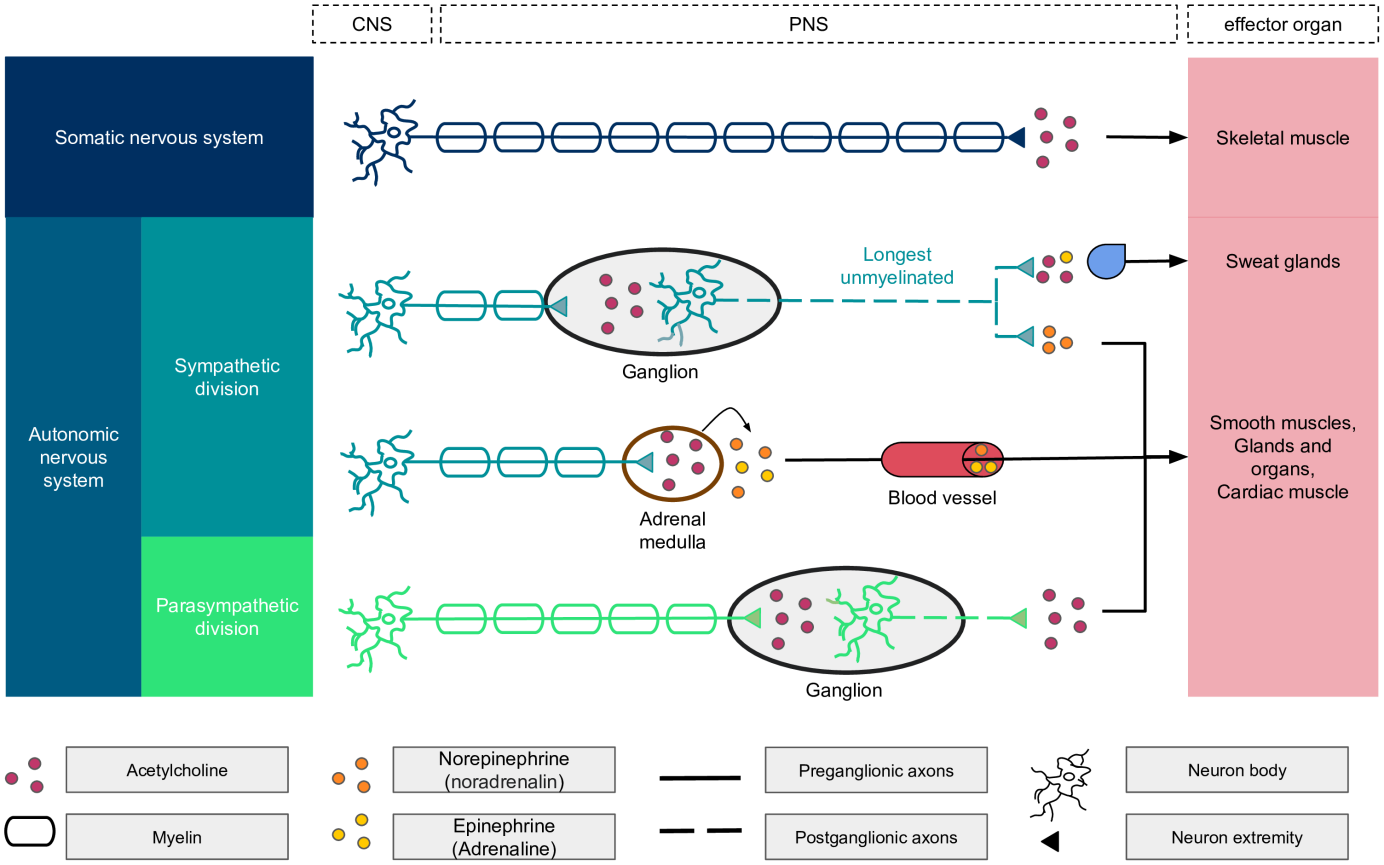
Details of the autonomic nervous system components with the central nervous system (CNS) and peripheral nervous system (PNS) components. The longest unmyelinated fiber of the body is found within the autonomic nervous system, the sympathetic division. At the end of sympathetic nerves, the neurotransmitter is epinephrine, except for the sudoral gland, which is activated by acetylcholine and epinephrine in a ratio of 80/20%. Acetylcholine binds to nicotinic receptors on neurons and muscarinic receptors on other biological structures.

Sudomotor function, which involves the regulation of sweating, is a specialized component of the Autonomic Nervous System (ANS—Figure 2) that is exclusively controlled by the sympathetic division (Gibbons, 2019). The sympathetic nervous system consists of a network of ganglia situated near the spine, which are connected to slender, long, unmyelinated C fibers that innervate the sweat glands. Notably, the fibers that innervate the sweat glands on the soles of the feet are the longest small nerve fibers in the human body, extending the entire length of the leg. These nerves are often the first to be affected by metabolic disturbances and other factors. Therefore, assessing sudomotor function in the soles of the feet can provide an early indication of the development of small fiber neuropathies.

**Figure 3:**
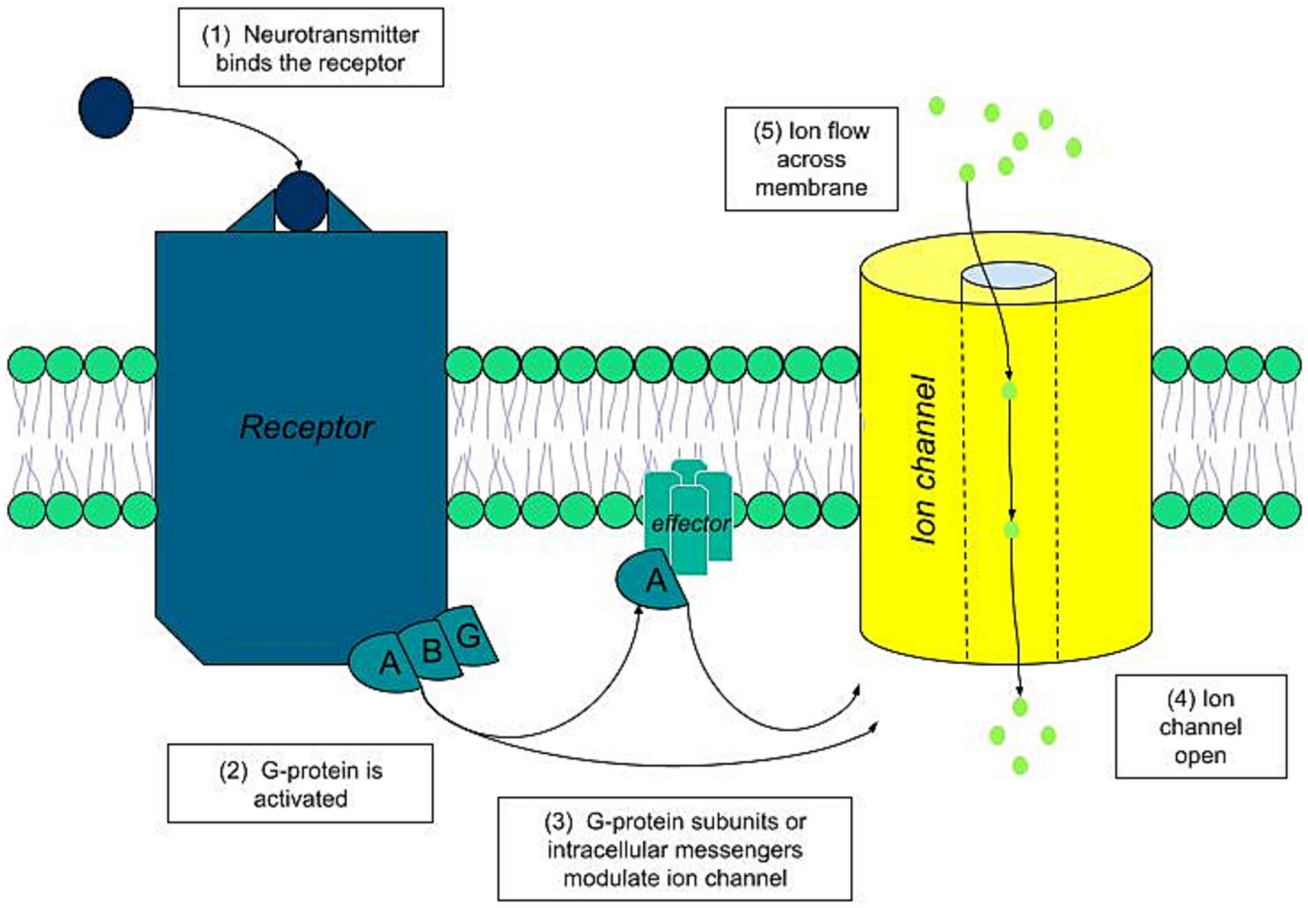
Cholinergic sequence activation.

Under normal physiological conditions, the activation of ESG begins with a chemical stimulus. In the cholinergic pathway, which is the most significant, this activation sequence involves the following steps (Cui and Schlessinger, 2015) (Figure 3):

- The neurotransmitter acetylcholine binds to its specific muscarinic acetylcholine receptor on the sweat gland cell membrane.
- This binding triggers the associated G proteins coupled to the receptor.
- The G proteins, or their secondary messengers, modulate the ion channels, leading to an ion flux across the luminal membrane.
- This activity generates polarization of the gland, resulting in voltages of around 10 millivolts.

While the small C nerves of sweat glands primarily utilize cholinergic neurotransmitters, a small proportion of adrenergic fibers are also present. The cholinergic fibers predominantly activate chloride ion channels through Muscarinic M3 receptors. In contrast, adrenergic fibers influence the Cystic Fibrosis Transmembrane Conductance Regulator (CFTR) chloride channels, which have been extensively studied in the context of cystic fibrosis.

In individuals with cystic fibrosis, the CFTR chloride channels, which are responsible for recycling chloride ions in the ducts (Figure 1d), are defective. This defect leads to an increased concentration of chloride ions in sweat, a hallmark of the disease. This characteristic is utilized in the diagnostic sweat test for cystic fibrosis, which measures the chloride ion concentration in sweat. The test induces sweating through pilocarpine iontophoresis, where an electrode is saturated with pilocarpine and a non-interfering electrolyte solution is placed on the skin. A mild electric current is then applied, driving the pilocarpine into the skin to chemically stimulate the sweat glands.

It is important to highlight that these small unmyelinated nerves possess the capacity for regeneration (Terenghi, 1995) with the rate of 0.177 ± 0.075 fibers/mm/day in healthy control subjects, while the presence of neuropathy was associated with a further reduction in regenerative rate (Polydefkis et al., 2004). Research has demonstrated that these nerves can regenerate more rapidly than other small sensory nerves, making them an optimal marker (Lauria et al., 2010) for gauging the effectiveness of therapeutic interventions and behavioral change (Gibbons et al., 2010; Illigens and Gibbons, 2013).

## Electrochemical skin conductance: historical concept and technical development

### Historical proof of concept in animal model

Vitale et al. (1986) have made a substantial contribution to scientific understanding through their research on the electrical stimulation of ESG extracted from the palms of adult monkeys. Their use of Electrical Field Stimulation (EFS) induced an immediate secretion from the glands, which promptly ceased once the stimulation ended. The secretion was found to be suppressed by atropine, a cholinergic antagonist, in a dose-dependent manner, indicating the involvement of cholinergic pathways.

However, a small portion of the glands did not respond to atropine, suggesting the presence of an atropine-resistant secretory mechanism. The study also found that physostigmine, which inhibits the breakdown of acetylcholine, enhanced the secretory response to EFS that was below the usual threshold for causing secretion. Crucially, lidocaine was able to completely and reversibly inhibit the secretion induced by EFS, yet it did not affect secretion triggered by methacholine, a cholinergic stimulant. This evidence supports the idea that electrical stimulation activates the nerve pathways responsible for delivering neurotransmitters that initiate sweat secretion. The research concluded that the primary activation of ESG in the palms is through cholinergic pathways and that EFS is a useful method for exploring the regulation of sweat secretion under both normal and pathological conditions.

The experimental setup for the electrical stimulation indicated that a voltage threshold between 750 millivolts and 1 volt is required to elicit a full secretory response from these ESG. This voltage range is critical for effectively activating the glands’ secretory function through nerve stimulation.

### ESC genesis

The genesis of the ESC was to (1) use the sweat’s electrochemical properties, (2) know sweat could be induced (e.g., chemical induction from work at Mayo Clinic), (3) but with an electric force (demonstrated in an animal model). The combination of this information led the first researchers, Phillippe Brunswich and Hanna Ayoub, to set up a technique using reverse iontophoresis (ion migration from skin to electrodes) and steady multiple chronovoltammetry, which is the application of a series of constant voltages, known as direct current voltages (or DC voltages). This approach also relies on a skin-specific property in which that skin is electrically insulated (essentially capacitive and negligible) at low voltage (less than 10 V). This has been demonstrated by Chizmadzhev et al. (1998).

### ESC device

**Figure 4:**
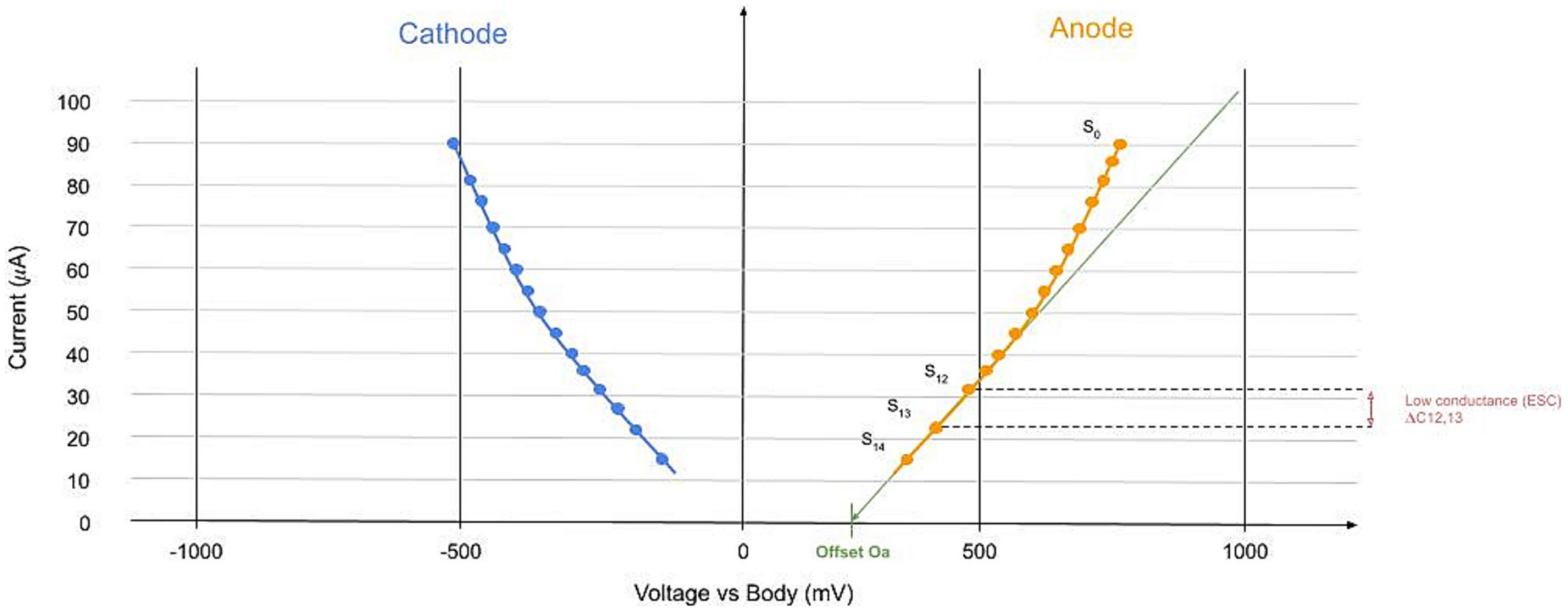
Example of the device-collected signal. In the X-axis, we represented the values of the tension applied. Each circle is a step, and in the Y axis, the current value is measured at the anode (orange) and cathode (blue). This signal is calculated for each limb. The main parameter is the linear slope at the low voltage of the I-V curve represented.

Sudoscan technology operates by applying a series of low DC voltages to the skin. Once the initial capacitive and transient effects settle, the skin’s electrochemical response is measured. This process involves a form of steady-state chronovoltammetry, which may be facilitated by reverse iontophoresis via the ESG, primarily involving chloride ions at the anode and protons at the cathode. In practical terms, a positively charged electrode known as the anode delivers a low direct current (DC) voltage to the skin, targeting the gland, while the current exits through the negatively charged electrode, the cathode (in Figure 4, we referred to this whole description as applied tension). The test is carried out on non-hairy (glabrous) skin areas, typically the palms and soles, where ESG are most concentrated [~500/cm^2^—Figure 2 from Taylor and Machado-Moreira (2013)].

**Figure 5:**
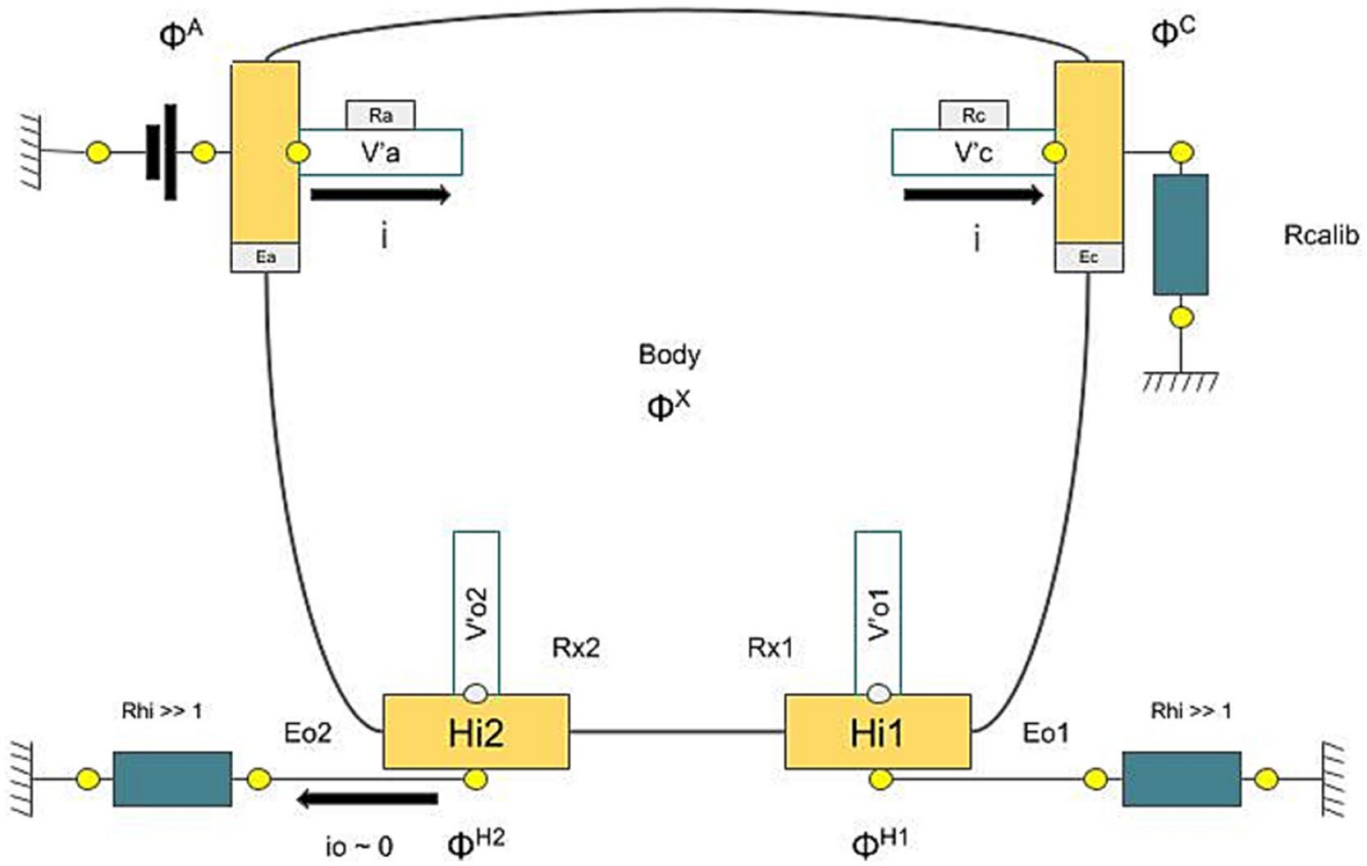
Electrical model of the Sudoscan device.

The device includes two pairs of electrodes for the hands and feet, which are linked to a computer that records and manages the data. During the test, patients place their hands and feet on these electrodes and remain still for the duration of the test, maintaining contact only with the electrodes. The device uses large stainless steel electrodes, which function alternately as an anode or cathode. At the low voltages applied, the skin’s outer layer (stratum corneum) acts as a capacitor, meaning the steady current passes only through the sweat glands. The active electrodes include the anode, where the voltage is applied, and the cathode, which is grounded through a calibration resistor to complete the circuit and measure the current. The other two electrodes, Hi1 and Hi2, are connected to the ground through high impedances, preventing current flow through them; they are used to measure the body’s voltage (Figure 5). The voltages ɸ^A^, ɸ^C^, ɸ^H1^, and ɸ^H2^ are measured from the current between the anode and cathode.

Immediately after the test, the electrochemical skin conductance (ESC) for all four extremities is displayed. The results are given in microsiemens (μS). The test also assesses asymmetry between the left and right sides of both hands and feet, highlighting potential differences in impairment (see ESC parameters section). No special preparation for patients, such as fasting or specific training for medical staff, is necessary to conduct a Sudoscan test. The procedure is quick, takes approximately 2 min, and is non-invasive. The cost of an exam is only a few dollars in general (depending on the device’s time in use over the years), making it very affordable.

### ESC modelisation

An electrochemical model of the measurement process was developed and is described in Supplementary File 1. This model explains the basis and assumptions underlying the physical laws in use. It provides the mathematical proof that ESC is not influenced by ion concentration, sweat conductivity, or the thickness of the outer skin layer (stratum corneum) under the assumptions made. This is why this technology serves as a non-invasive, *in vivo* method for the immediate determination of surface conductances of both the excretory and secretory portions of the sweat glands. It also demonstrates a connection between sudomotor function and the electrical properties of the glands, such as capacitance (C) and conductance (G), and relates these to the gland’s structure. This includes the muscarinic and adrenergic innervation quality and the various ion channels, pumps, shunts, and co-transporters present.

Notably, it is essential to standardize the conductance values to enhance the accuracy of the results. Skin conductance can vary widely, from just a few microsiemens (μS) to several hundred μS, and for a circuit of conductance 1 microS, the direct current traversing it increases by 1 microA for every increase of 1 V of the potential difference across the circuit. The purpose of this standardization is to compress the range of high and healthy conductance values while simultaneously expanding the range of low and potentially indicative conductance values (see Validation—Clinical Development). For a raw conductance value measured in microsiemens, the standardization process involves a function that transforms the conductance range from 0 to infinity (0, ∞) into a number between 0 and 100.

### ESC main parameter

For each limb (hand or foot) and on each side (left or right), the raw signal collected is a current–voltage (I-V) curve with specific properties related to the human body (Fish and Geddes, 2009). I is current (the same current going through the anode and cathode), and to measure this current, we use the traditional way of I=U/R. R is the standard resistance between the cathode and the earth. For each voltage step defined, the current is collected at the anode and the cathode. This approach allows us to build the I-V curve, resulting in data as presented in Figure 4. The main parameter is the low conductance LC. The ESC is calculated between voltage steps 12 and 13 (see Skin Conductance: Gland Wall Ion Permeability in Supplementary File 1). The ESC is defined between steps 12 and 13 because of the overpotential and the offset (Lair et al., 2019) (see other parameters in Supplementary File 1) and because this low voltage part is linear. It is given by the discrete slope (here at the anode and similar at the cathode):


LC=(S12,S13)=I13−I12∅−12A∅+13A∅−12X∅,13X

where $\emptyset^{A}$ is the voltage at the anode, $\emptyset^{X}$ the voltage at the cathode and *I* the body potential measured at the different voltage steps. From this, the averaged value on feet called feet ESC (ESC_F_), the hands ESC (ESC_H_), feet asymmetry (ESC_FA_), hands asymmetry (ESC_HA_), and ratio HF (ESC_RHF_) are calculated.

## Electrochemical skin conductance: validation and clinical development

Since there was no pre-existing device with proven clinical effectiveness for this type of sudomotor testing, Sudoscan technology had to undergo evaluation through clinical trials. The test’s quick and non-invasive nature, which stands in contrast to most clinical and research tests for small fiber neuropathy (SFN) diagnosis, allowed for its integration as an additional assessment during routine patient follow-ups.

### ESC healthy values and accuracy

Normal electrochemical skin conductance (ESC) values for adults were established in a study involving more than 1,350 healthy individuals (Vinik et al., 2016). This study serves as a foundation for normative values. From this study, it was established that the average ESC values for both women and men at the hands (75 [57–87] vs. 76 [56–89] μS, *p* = 0.35) and feet (83.5 [71–90] vs. 82.5 [70–91] μS, *p* = 0.12) did not show significant differences. Overall, factors such as body mass index (BMI) and exercise habits had no notable impact on ESC; there was a very slight decrease in ESC with advancing age and a noticeable variation based on race/ethnicity. These findings were corroborated by a study within a healthy Indian cohort (Shivaprasad et al., 2018) and a diabetes Chinese cohort (Mao et al., 2017). Furthermore, Sudoscan tests conducted on 100 healthy children (Leclair-Visonneau et al., 2016) indicated that their normal values align with adult values, although the sample size of 100 children is considered small for establishing definitive normal values, indicating a need for further research in this area.

The method’s precision was assessed following Food and Drug Administration (FDA) guidelines, which involved conducting two measurements on each of three different devices, totaling six Sudoscan tests per individual. This evaluation revealed a coefficient of variation for feet/hand ESC of 4% in healthy subjects and 7% in diabetic patients (Bordier et al., 2016). These study outcomes demonstrate that Sudoscan technology is reliable across diverse clinical settings and population groups. Moreover, its consistent performance over time suggests that any observed changes in ESC are indicative of alterations in sudomotor function, warranting additional clinical evaluation.

### Performance versus intraepidermal nerve fiber density

The recognized benchmark for diagnosing distal small fiber neuropathy is the measurement of intraepidermal nerve fiber density (IENFD). IENFD testing is a histological technique used to quantify the density of nerve fibers within the epidermis. The process involves taking a small skin biopsy, typically from the lower leg, which is then fixed, embedded, and sectioned for staining with specific antibodies that highlight the small nerve fibers. The stained skin sections are examined under a microscope, and the number of nerve fibers crossing the dermal-epidermal junction is counted. This count is then normalized to the length of the epidermal surface to calculate the nerve fiber density. The procedure is invasive and requires local anesthesia, and the biopsy sample must be processed by a laboratory with expertise in this specialized staining technique (Smith et al., 2005; McArthur et al., 1998; Thomas et al., 2023).

Research has been conducted to compare the diagnostic accuracy of Sudoscan and IENFD in both healthy individuals and patients undergoing evaluation for small fiber neuropathy at specialized centers. Smith and colleagues found that the area under the receiver operating curve (ROC), commonly called the area under the curve (AUC), for Sudoscan’s electrochemical skin conductance (ESC) and IENFD were comparably effective (0.761 and 0.752, respectively) (Smith et al., 2014). Novak and colleagues also found a strong correlation between ESC and IENFD when adjusted for body weight (correlation coefficient of 0.73, with a significance level of *p* = 0.0001) (Novak, 2019). These studies indicate that Sudoscan could serve as a reliable, non-invasive alternative for diagnosing small fiber neuropathy, offering immediate results without discomfort. Furthermore, Sudoscan facilitates easier and more patient-friendly repeated assessments for monitoring treatment efficacy or disease progression compared to skin biopsies.

### Performance versus other methods

#### QSART

Although it is generally only available in specialized medical facilities, the Quantitative Sudomotor Axon Reflex Test (QSART) is probably the most accessible test for assessing sudomotor function and, as a result, is the most frequently used in clinical practice (Illigens and Gibbons, 2009). Sudoscan has been evaluated against QSART in two separate studies, which demonstrated that Sudoscan had a higher Area Under the ROC Curve (AUC) (0.77 vs. 0.57, 0.71 vs. 0.53 when compared to QSART, respectively) in diagnosing small fiber neuropathy and diabetic neuropathy (Krieger et al., 2018; Callaghan et al., 2018) based on already characterized patients. While QSART is an established and validated measure of sudomotor function, it has been found to have certain diagnostic limitations when it comes to small fiber and diabetic neuropathies. Here are several potential limitations to QSART. Firstly, QSART can be expensive. Additionally, the iontophoresis of acetylcholine used to induce sweating may confuse pharmacy staff unfamiliar with this technique. Proper control of ambient temperature and humidity during testing is crucial. Results must be interpreted considering both age and gender. Furthermore, medication use, topical moisturizers, and hydration status can lead to inaccurate results (Illigens and Gibbons, 2009; Illigens and Gibbons, 2019).

#### Neuropad^®^

At the date (end of 2024), the only technology provoking the same biological process as the Sudoscan was the Neuropad^®^ (Bilen et al., 2007; Papanas et al., 2007). It is a simple, quick, and inexpensive approach like Sudoscan, but it has one major drawback: it is not a quantitative measure since it is based on colorimetry from sweat. One study established the higher performance of Sudoscan as compared to Neuropad^®^ (Zouari et al., 2019), but since Neuropad^®^ is not a quantitative method, it should be compared contextually.

#### CNFL

There is a notable correlation between Sudoscan’s ESC and corneal nerve fiber length (CNFL), as determined by *in vivo* confocal microscopy (with a correlation coefficient squared (R^2^) of 0.8) (Rousseau et al., 2016). CNFL is a proxy indicator for nerve damage in conditions like diabetic sensorimotor and autonomic neuropathy. In a particular study focusing on patients with transthyretin familial amyloid polyneuropathy—a genetic condition characterized by small-fiber neuropathy—the researchers found a very strong correlation between ESC and CNFL in a small group of patients (*n* = 15) with varying degrees of neuropathy severity. It is important to note, though, that confocal microscopy is a complex procedure that requires sophisticated eye equipment, specialized software, and the application of an anesthetic to the cornea before examination.

#### LEP, QST, SSR, and CDT

In comparison with four reference diagnostic methods for small fiber neuropathy, Sudoscan was found to be less sensitive than Laser Evoked Potential (LEP), a highly specialized and time-consuming research method; however, Sudoscan had marginally better diagnostic performance than Quantitative Sensory Testing (QST) for warm detection and much better performance than cold detection threshold (CDT) and sympathetic skin response (SSR) (Lefaucheur et al., 2015).

### Diabetic peripheral neuropathies evaluation

Studies in Chinese, Mexican, and American patients have consistently demonstrated the test’s ability to detect DPN with high sensitivity and specificity (Casellini et al., 2013; Selvarajah et al., 2015; Mao et al., 2017; Smith et al., 2014; Krieger et al., 2018; Jin et al., 2018; Yajnik et al., 2012; Carbajal-Ramírez et al., 2019; Gin et al., 2011) while correlating well with clinical signs and symptoms of neuropathy, making it a valuable screening tool.

### Nerve regeneration follow-up

Another interest of the ESC is the ability to follow up on small nerve regeneration and thus provide an objective measure of specific treatment, drugs, or lifestyle change. Some publications (Casellini et al., 2016; Didangelos et al., 2021; Yajnik et al., 2019) paved the way for the study of it, but more are needed to understand the regeneration speed and process within specific groups of patients.

### Proposed threshold—values range

The normative healthy studies and diabetic studies led the researchers to define several thresholds and propose a healthy/unhealthy range of values:

- Under 50: unhealthy values
- Between 50 and 70: moderately healthy values
- Over 70: healthy values
- For Chinese, Indian, and African-American, thresholds are minored by 10 at the foot (Vinik et al., 2016), and for the hand, it is minored by 10 only in the African American ethnic group.

We suppose that the sweat gland density and sweat chemical properties could be related to genetic factors, but no studies have been conducted in that field in general. Medical patient history and current condition should always be used when assessing any SFN diagnostic to define the precise condition that originates from small nerve degradation. In general, lower ESC values are associated with a higher frequency of neuropathy signs; see results from Novak (2019) (Utah Early Neuropathy Scale—UENS R = 0.388, *p* < 0.004—and Michigan Neuropathy Screening Instrument—MNSI R = −0.398, *p* < 0.005 -).

## Discussion

### Differences with electrodermal activity (EDA or GSR/SSR)

Electrochemical skin conductance (ESC) is often confused with measures of physiological responses such as the sympathetic skin response (SSR) (Arunodaya and Taly, 1995), galvanic skin response (GSR) (Nayak et al., 2023; Polat and Özen, 2023), or electrodermal activity (EDA) (Klimek et al., 2023; Anmella et al., 2024). However, these measures are fundamentally different, necessitating unique analytical methods and interpretive contexts. EDA, which we will use as an umbrella term for these related measures (*please note that the 2023 legal FDA term in use is GSR under the code GZO*), quantifies the fluctuation in skin conductance in reaction to any stimuli or arousal events (talk, stress, fear, pain, pleasure, joy, …).

EDA is a valuable tool in psychological, neurological, and emotional research, as it helps to understand the body’s response to various stimuli, such as physical contact, auditory cues, visual stimuli, or even speech (Kucera et al., 2004; Cox et al., 2023). The sensitivity of EDA measurements depends on the precise placement of electrodes on the skin and requires careful calibration and specific analytical tools for interpretation (Posada-Quintero and Chon, 2020; Singaram et al., 2023). EDA has been widely used in applications such as lie detection and stress analysis, where physiological responses to questioning or stressful situations are of interest (Rahma et al., 2022; Matton et al., 2023).

The recent surge in the popularity of wearable technology has brought EDA measurements to the forefront of personal health monitoring. For example, Google’s integration of continuous electrodermal activity (cEDA) into its Pixel Watch 2 represents a significant step in consumer health technology. Google’s approach combines EDA readings with a machine learning algorithm that also considers heart rate, heart rate variability, and skin temperature to provide insights into the wearer’s stress levels (Google, 2023) (clinical studies are needed to evaluate it). Other wearables like the Oura, Samsung, and Helio ring also use the EDA but with no medical claim (in date of mid-2024) that could be found in the FDA 510 k database or clinical trial database.

In contrast, ESC is not a continuous monitoring tool but rather a diagnostic test that challenges the sudomotor system, which is responsible for sweat production. EDA measures passive skin conductance, whereas ESC is an active measure that comes from local, controlled, and direct sweat gland stimulation, which is a big difference. By applying a specific voltage through electrodes made of particular materials, ESC can assess the integrity and function of small nerve fibers that innervate sweat glands. This test is particularly useful in the medical field for diagnosing conditions such as small fiber neuropathy, where the performance of the sudomotor system is indicative of disease presence and progression.

Currently, a scientific question arises. Does the EDA measure passive sweat conductance or passive skin conductance? In the case of ESC, experiments were conducted with chemicals to stop sweat production and define the process (see *Historical Proof of Concept in Animal Model*). To our knowledge (in 2024), no publications made the experimentation with the equipment used to measure EDA on that point. It could be an interesting fundamental study to perform since EDA has started to be widely integrated into daily digital devices.

In summary, while EDA and ESC both involve skin/sweat conductance, they serve different purposes, are interpreted in vastly different contexts, and need different analytical tools. A quick way to determine the type of measure is to look at their value in microsiemens, which typically range from 0 to 5 for EDA and generally 10 to 90 for ESC. EDA is a passive continuous signal of the autonomic nervous system’s response to stimuli and is used extensively in psychological and emotional research, as well as in consumer health technology for stress management. ESC, on the other hand, is an active single-measure diagnostic tool used in medical settings to evaluate the function of the sudomotor system and diagnose neuropathic conditions. Each has its own set of protocols, equipment, and significance, highlighting the importance of distinguishing between these measures in both clinical and consumer applications.

### Technology’s future

A major topic of research is to exploit all the information contained in the current voltage curve, not just its slope at low voltages. Several parameters can be defined to characterize this curve (see Others parameters part from Supplementary File 1). They can be useful in two ways: (1) replace the main parameter where it is not useful/validated, or (2) bring more granularity in already known cases (e.g., diabetes).

Recently, Withing integrated the ESC measures into two of its scales (BodyComp and Body Scan^@™^) after a partnership with Impeto. The company published a clinical study demonstrating the measure agreement between its scales and the Sudoscan devices (Riveline et al., 2023) with mostly perfect correlation. The only difference is that the scales take measure on feet only. This point is important and should be considered when choosing a device for any case. Thus, longitudinal ESC data are collected from users around the globe. Notwithstanding the challenges posed by real-life data analysis, it opens doors for new research. In particular, it brings the possibility of analyzing individual time series or large tendencies. It will also be interesting to see if normal values can be validated or fine-tuned at a large scale to improve disease threshold specificity.

## Conclusion

In conclusion, peripheral neuropathies (PNs) encompass a diverse group of disorders affecting the peripheral nervous system, with symptoms and severity varying across cases. These conditions can impact motor, sensory, and autonomic nerve fibers, leading to a wide array of clinical manifestations. Diagnostic techniques such as nerve biopsies and skin biopsies have significantly improved our ability to identify and understand these neuropathies, particularly small fiber neuropathies.

The introduction of the Quantitative Sudomotor Axon Reflex Test (QSART) represented a notable step forward in objectively assessing peripheral autonomic neuropathies (PANs), despite its limitations in clinical application. The field has evolved further with the development of ESC by Impeto Medical, a non-invasive, affordable, reproducible method that has gained FDA clearance and is increasingly used in medical research and practice. The integration of ESC technology into Withings’ consumer-grade Body Scan^@™^ scale represents a significant leap forward, enabling at-home monitoring and the generation of large datasets for research, potentially transforming the management of neuropathic conditions.

This historical and conceptual review has explored the biological and physical principles underlying ESC and its distinction from other sudomotor tests, aiming to provide a comprehensive reference for medical professionals. The unique properties of eccrine sweat glands (ESG) and their regulation by the autonomic nervous system highlight the importance of sudomotor function as both a diagnostic and therapeutic target. The capacity for regeneration in small unmyelinated nerves offers hope for monitoring and improving treatment outcomes in peripheral neuropathies. As the field continues to advance, there remains immense potential for new discoveries and improved patient care in the management of peripheral neuropathies.

## Limitations

As with other biomarkers for PANs, ESC is not disease-specific but serves as a valuable screening tool. It provides general insights into the health of peripheral nerves, defining the degree of sudomotor dysfunction or imbalance within the sympathetic/parasympathetic system. Therefore, it should be followed by a clinical examination to understand the patient’s medical history and context for an accurate diagnosis. Additionally, there is limited data on the effects of medications on ESC in the literature. The impact of cholinergic medications, narcotics, caffeine, and other substances should be further studied, particularly in real-world measurement scenarios.

## Footnotes

^1^ http://creativecommons.org/licenses/by-nc-nd/4.0/

## Author’s note

This paper is a rework and update of a previous communication (Chapter 6 : A Simple and Accurate Method to Assess Autonomic Nervous System through Sudomotor Function, advances in biosensors review, volume 3, 2020) that had been published in a book chapter but had never gone under scrutinous peer review process. In an effort to make the theory behind Electrochemical Skin Conductance (ESC) more accessible we decided to update this work and transform it into a proper scientific publication.
