# Supplementary material for: From Sudoscan to bedside: theory, modalities, and application of electrochemical skin conductance in medical diagnostics
Source: Front Neuroanat. 2024 Oct 23;18:1454095. doi: 10.3389/fnana.2024.1454095 (PMC11551929; doi:10.3389/fnana.2024.1454095)
Supplement: Supplementary file 1 [file Data_Sheet_1.PDF]

Supplementary file

Table of contents

Table of contents....1
Mathematical Model of Eccrine Gland.... 2
1. Geometrical Model of Eccrine Gland, Variables and Currents....2
    1.1 Geometry and Variables....2
    1.2 The Currents.... 3
    1.3 Ohm's Law and Electrical Field.... 4
2. Governing Conservation Equations....5
    2.1 Mass Conservation....5
    2.2 Momentum Conservation.... 7
    2.3 Momentum Steady Equation: Concentration Constancy....9
    2.4 Mass Steady Equation....11
3. Steady State additional analytics for the Conservation of Mass Equation.... 12
    3.1 Continuity.... 12
    3.2 The Voltage inside the Gland is Almost Constant.... 13
    3.3 The Axial Current is Almost Piecewise Linear....15
4. Skin Conductance: Gland Wall Ion Permeability....17
5. Normalization.... 18

Others parameters.... 18
Figures.... 21
Figure 1.... 21
Figure 2.... 22
Figure 3.... 22
Figure 4.... 23
Figure 5.... 23
Tables.... 24
Table 1....24
Bibliography.... 25

Mathematical Model of Eccrine Gland

1. Geometrical Model of Eccrine Gland, Variables and Currents

1.1 Geometry and Variables

The eccrine gland is composed of two parts: the secretory portion, a coil where physiologically the sweat is (isotonically) filtered from the interstitium; and the excretory portion where some species can move in both directions (entry or absorption according to their electrochemical gradient) across some ionic channels or not. The excretory portion is a quasi-straight duct that leads to a pore on the skin surface. These two regions have lengths of the same order, with the coil being slightly larger than the duct. The geometrical model consists in unrolling the coil and joining it to the duct to form, as in the Chizmazdhev model (Chizmadzhev et al., 1998), a cylindrical tube, but here of a finite and more realistic length (Figure 1).

The principal variables are the concentrations c_k and the velocities u_k of the electro-active ions present in the sweat: $k \in S = \{Cl^-, H^+\}$. We shall add the electric potential inside the gland Φ. It will be expressed from the principal variables, see further. They are all functions of (x, t), where x is the abscissa along the axis and t is time. The geometric parameters are:

- h: thickness of the stratum corneum (SC),
- r_e: radius of the duct, r_s: radius of the coil,
- Lengths L_e, L_s of excretory and secretory portions.

The electrical parameters are:

- σ: conductivity of the electrolyte (sweat),
- Φ^A: potential applied at the anode,
- Φ^{ext}: potential reached by the body after the application of the anodic tension. To simplify the analysis and the calculations, the potential $\Phi^{ext} = 0$ will be chosen as reference.

Surfacic conductances of the wall of the two portions, G_k^e, G_k^s, depend on the species k and are generally a function of the potential difference between both sides of the gland wall $(\Phi - \Phi^{ext})$. They may involve the gaping probabilities of some ionic channels and/or electroporation phenomena (Chizmadzhev et al., 1998). Surfacic capacitances of the wall of the two portions, C_k^e, C_k^s, may depend on the species k and are almost constants, according to Chizmazdhev (Chizmadzhev et al., 1998).

1.2 The Currents

A. The cross-wall current is a transverse current due to charges that cross the wall of the gland, and which depends on the ion *k*. Its density (per unit area on the wall of the gland) depends on the way the crossing occurs, through an ion channel or not. The ion channel is related here to the Cl^- ion that can go through this epithelial membrane using its own dedicated ion channel (Chen and Miller, 1996; Wills and Fong, 2001; Granger et al., 2003). This ion channel approach is more faithful than a simple conductance model because it also considers the chemical gradient. The density of this current J_k^{cross} for ion k is given by :

$$J_k^{cross} = z_k . G_k . \left(\Phi - \Phi^{ext} - \Phi_k^{Nernst}\right), \qquad 1.2.0$$

where z_k is the valence of the ion and G_k the conductance per unit area, Φ_k^{Nernst} is the membrane equilibrium potential that exactly opposes the net diffusion of the ion *k* through the wall. It is given by Nernst's law (Cronin, 1981) (Figure 2)

$$\Phi_k^{Nernst} = \frac{R.T}{z_k.F} . ln\left(\frac{c_k}{c_k^{ext}}\right), \qquad 1.2.1$$

in which R is the perfect gas constant, T is the absolute temperature and F the Faraday constant (charge of a mole). In physiological polarizations, this channel behaves according to the Boltzmann function, voltage dependent.

Here, it is important to mention that the muscarinic channels are driven by cholinergic neurotransmitters, which are supposed to be voltage dependent. The duct, meanwhile, consists essentially of CFTR channels that are minimally voltage dependent and especially sensitive to the chemical (concentration) gradient. Thus the CFTR channels are not taken into account within the modelisation at low voltage.

B. The capacitive wall current is a transverse current due to charges that accrue on (or leave) the wall of the gland; it may depend on the ion *k* and its density (per unit area on the wall of the gland) and is expressed as follows:

$$J_k^{capa} = z_k . C_k^p . \frac{\partial \Phi}{\partial t}, \; p \in \{s, e\}, \qquad (1.2.2)$$

where C_k^x is the capacitance per unit area, which is almost constant ~$0.5\ \mu F/cm^2$ [12]. This current vanishes in steady states.

C. The axial current is a current along the gland axis *x* due to the motion (migration) of ions following this axis inside the gland. The surfacic density of the axial current, as a function of the main variables, for any species k, is by definition:

$$J_k^a = F.z_k.c_k.u_k, \tag{1.2.3}$$

Then the axial current is given by:

$$I_k^a = \pi.r^2.J_k^a, \tag{1.2.4}$$

and the total density and current are:

$$J^a = F.\sum_k z_k.c_k.u_k, \qquad I^a = \pi.r^2.F.\sum_k z_k.c_k.u_k \tag{1.2.5}$$

1.3 Ohm's Law and Electrical Field

Ohm's law $J = \sigma E =- \sigma\frac{\partial\Phi}{\partial x}$ integrated over the section of the tube gives

$$I^a = -\sigma.\pi r^2\frac{\partial\Phi}{\partial x} \tag{1.3.1}$$

The electric field *E* is inferred there from:

$$E\equiv -\frac{\partial\Phi}{\partial x} = \frac{F}{\sigma}\sum_k z_k.c_k.u_k \tag{1.3.2}$$

2. Governing Conservation Equations

It is shown in the following that the whole problem can be solved sequentially: first the mass equation will lead to the potential, then the momentum balance gives the constant concentration, and finally the velocity can be deduced from Ohm's law.

2.1 Mass Conservation

Let us consider a continuous medium where ϱ is the density or volumic mass and $\underline{U}$ is the velocity, in a material volume $\Omega(t)$, *i.e.* a subdomain of the medium, of arbitrary size, composed of the same particles, dependent of time t, that we will study and follow in his motion, of boundary $\partial\Omega(t)$ with its outward normal $\underline{n}$. We note the spatial coordinate (position) $\underline{x} \in \Omega(t)$. Let $a(\underline{x}, t)$ be a scalar or vector field regular enough. We recall the transport theorem:

$$\frac{d}{dt} \int_{\Omega(t)} a(\underline{x}, t)\ d\Omega(t) \ = \ \int_{\Omega(t)} \left[\frac{\partial a}{\partial t} + div(a \otimes \underline{U})\right] d\Omega(t), \tag{2.1.1}$$

$\otimes$ designates the tensor product, $\frac{d}{dt}$ is the (total or particular) time derivative, and $\frac{\partial}{\partial t}$ is the partial time derivative. In addition, for a vector field $\underline{f}$ regular enough, one can prove the divergence formula:

$$\int_{\partial\Omega(t)} \underline{f}.\underline{n}\, d\Sigma(t) \ = \ \int_{\Omega(t)} div(\underline{f}) d\Omega(t) \tag{2.1.2}$$

A balance equation for the quantity ($\varrho\ a$) can be written under the general form:

$$\frac{d}{dt} \int_{\Omega(t)} \rho\, a\ d\Omega(t) \ = \ \int_{\Omega(t)} A\, d\Omega(t) + \int_{\partial\Omega(t)} A.\underline{n}\, d\Sigma(t), \tag{2.1.3}$$

where A is a (volumic) production-disappearance term and Γ is a (surfacic) exchange flux across the boundary $\partial\Omega(t)$. Using the transport theorem (2.1.1) and the divergence formula (2.1.2), we get immediately:

$$\frac{d}{dt}(\rho\, a) + div(\rho\, a \otimes \underline{U}) \ = \ A \ + \ div(\Gamma) \tag{2.1.4}$$

For a one dimensional problem, this reduces to

$$\frac{\partial}{\partial t}(\rho\, a) + \frac{\partial}{\partial x}(\rho\, a\, u) \;=\; A + \frac{\partial \Gamma}{\partial x}\,, \qquad (2.1.5)$$

The conservation of mass is obtained by setting to $a = 1$ and $\Gamma=0$ (by definition of the material volume $\Omega(t)$):

$$\frac{\partial}{\partial t}(\rho) + \frac{\partial}{\partial x}(\rho\, u) \;=\; A \qquad (2.1.6)$$

It remains now to specify the source term A which represents, per unit volume, the loss or gain due to the transverse (cross-wall and capacitive) current. We will describe briefly the mass equation source term. First, recall that for ion k, the density ρ_k is related to our variable: its concentration c_k by the simple relation :

$$\rho_k \;=\; c_k .\, M_k, \qquad (2.1.7)$$

where M_k is the “constant” molar mass. We will calculate A on a slice inside the gland with radius r and thickness dx. Its volume is $V \;=\; \pi.\, r^2.\, dx$ and its lateral area is $S \;=\; 2\pi.\, r.\, dx$; the total current crossing this lateral surface is :

$$-\; S.\, J_k^t, \qquad (2.1.8)$$

where $J_k^t \;=\; J_k^{cross} + J_k^{capa}$. So if we note e the elementary charge and m_i the mass of the ion, the mass transfer is :

$$-\; S.\, J_k^t.\frac{m_k}{e} \equiv -\; S.\, J_k^t.\frac{M_k}{F} \qquad (2.1.9)$$

Hence, per unit volume :

$$A \;=\; -\; S.\, J_k^t.\frac{M_k}{F}.\frac{1}{V}, \qquad (2.1.10)$$

Finally, with the concentration, the mass balance is given by:

$$\frac{\partial c_k}{\partial t} + \frac{\partial (c_k.u_k)}{\partial x} = \frac{-2}{r.F}.J_k^t, \qquad (2.1.11)$$

where $J_k^t = J_k^{cross} + J_k^{capa}$. This is recognized as a classical transport equation with an original source term. Thus, by multiplying (2.1.11) with z_k, using (1.3.1) and (1.3.2), the (total) axial current is written:

$$\pi.r^2.F\frac{\partial\left(\sum_k z_k.c_k\right)}{\partial t} + \frac{\partial I^a}{\partial x} = -2\pi.r.\sum_k z_k.J_k^t, \qquad (2.1.12)$$

which at steady state ($\partial/\partial t = 0$) shows that the variation of the axial current equalizes the sum of the transverse currents.

Now as $J_k^t = J_k^{capa} + J_k^{cross}$, by using expression (1.2.2) of J_k^{capa} and Ohm's law (1.3.1) with the capacitive currents gathered, we end up with:

$$\frac{2F}{r}\frac{\partial\left(\sum z_k.c_k\right)}{\partial t} + \left(\sum_k C_k\right)\frac{\partial\Phi}{\partial t} = \frac{\sigma.r}{2}\frac{\partial^2\Phi}{\partial x^2} - \sum_k z_k.J_k^{cross} \qquad (2.1.13)$$

To compare to the purely electric balance equation (which is the unique equation within Chizmazdhev's model) of Chizmazdhev (Chizmadzhev et al., 1998) chi: eq. (8) page 846, in which the first term does not exist! See the extract in Figure 3.

2.2 Momentum Conservation

We can define the classical conserved physical quantities are: Mass, momentum and energy. The last one is of less interest here because it introduces thermic power, heat exchanges and additional variables of at least the temperatur. Moreover it will not bring anything here because the measure is isotherm. The two others are summarized in Table 1. Using equation (2.1.5) and Table 1 demonstrate that the momentum conservation is written:

$$\frac{\partial}{\partial t}(\rho\, u) + \frac{\partial}{\partial x}\left(\rho\, u^2\right) = F \qquad (2.2.1)$$

It remains now to specify the source term F which represents, per unit volume, the resultant of the present external forces. In the following, and since only one species is involved at each electrode side, the species index i will be dropped. The ions are supposed to be rigid spheres moving in a continuous incompressible fluid. Recall that when some charged species are in motion, they create an electric field E (already seen) and a magnetic one B. Assuming that thermal agitation and interactions between species and with the wall are negligible and that Stokes law(Layton, 1975) is applicable, the species is submitted to the following forces:

Lorentz force:

$$z.e.E + z.e.u \wedge B, \qquad (2.2.2)$$

the second term is rigorously null in the 1D model because it is orthogonal to the velocity.

Drag force due to sweat opposition:

$$-\ \xi.(u - v), \qquad (2.2.3)$$

where v is the speed of sweat (here $v = 0$, because the application of the electrode firmly against the skin plugs its gland pores and blocks the physiological sweat flow) and ξ is the Stokes coefficient (Layton, 1975) given by :

$$\xi = 6\pi.\mu.H, \qquad (2.2.4)$$

where μ is the dynamic viscosity of the sweat (water) and H the hydrodynamic radius of the ion. Stokes law is only approximative because the shape of the ions is certainly not spherical. Moreover, at a microscopic scale, the ions bathe in a medium far from being continuous but rather filled with particles of similar size to one of the ions studied. However, the hydrodynamic radius is the radius of a hypothetical hard sphere that diffuses with the same speed as the ion. And in fact, deduced from the real mobility of the ion in the electrolyte, defined by:

$$M \equiv limu/E = (z.e)/\xi, \qquad (2.2.5)$$

which was tabulated, see for example Atkins & al (Atkins and Paula, 2017). Thus, the resultant force is:

$$R = z.e.E - \xi.u \qquad (2.2.6)$$

And per unit volume:

$$F = R.\frac{\rho}{m} \equiv R.\frac{c.M}{m} \quad (2.2.7)$$

We infer finally the momentum conservation law:

$$\frac{\partial(c.u)}{\partial t} + \frac{\partial\left(c.u^2\right)}{\partial x} = \frac{z.e}{m}.c.E - \frac{\xi}{m}.c.u, \quad (2.2.8)$$

2.3 Momentum Steady Equation: Concentration Constancy

A numerical solution of the whole problem, i.e., the previous partial differential equations augmented with suitable initial and boundary conditions, although it may be delicate, is possible. Here it is conceivable to simplify the problem to obtain analytical results in two ways:

- Considering steady solutions, which allow capacitive aspects and time derivatives to vanish,
- Considering only electroactive ions, i. e., Cl^- at the anode and H^+ at the cathode.

At steady state, the equation for the momentum, for chloride near the anode, reduces to:

$$\frac{d(c.u^2)}{dx} = -\frac{e}{m}.c.E - \frac{\xi}{m}(c.u) \quad (2.3.1)$$

A main result is the theorem of constant concentration:

Assuming that *c* is constant then

$$c_* \leq c \leq c_*.(1 + \alpha), \quad (2.3.2)$$

with

$$\alpha = \frac{8\, m.e.G.\Phi^A}{\xi.\left[r.\xi.\sigma + \sqrt{(r.\xi.\sigma)^2 + 16\, \sigma.m.e.r.G.\Phi^A}\right]}, \quad (2.3.3)$$

$$c^* = \frac{\xi.\sigma}{e.F},$$

$$C = \frac{\sigma^2.m}{F^2.e}$$

Next we will define the result proof (i). At steady state, the equation for the momentum, for chloride near anode, reduces to :

$$\frac{d(c.u^2)}{dx} = -\frac{e}{m}.c.E - \frac{\xi}{m}(c.u), \tag{2.3.4}$$

which, by using the expression of the electric field (1.3.2)

$\left(E = -\frac{d\Phi}{dx}\right)$ and Ohm's law (6.3) $\left(c.u = \frac{\sigma}{F}.\frac{d\Phi}{dx}\right)$ gives:

$$\frac{\sigma^2}{F^2}\frac{d\left(\left[\frac{d\Phi}{dx}\right]^2.\frac{1}{c}\right)}{dx} = \frac{e}{m}.c.\frac{d\Phi}{dx} - \frac{\xi.\sigma}{m.F}.\frac{d\Phi}{dx}, \tag{2.3.5}$$

or else, after developing and simplifying by $\frac{d\Phi}{dx}$:

$$c = c^* + C.\left[\frac{d\Phi}{dx}.\frac{d}{dx}\left(\frac{1}{c}\right) + \frac{2}{c}.\frac{d^2\Phi}{dx^2}\right], \tag{2.3.6}$$

$$c^* = \frac{\xi.\sigma}{e.F},$$

$$c = \frac{\sigma^2.m}{F^2.e},$$

To prove, begin with equation (2.3.6) above and suppose *c* is constant, then we have $c'(x) = 0$ and

$$c = c^* + \frac{2C.\Phi''}{c} \Leftrightarrow c^2 - c^*.c - 2C.\Phi'' = 0 \tag{2.3.7}$$

Solving this second order algebraic equation, we get the constant c^* and an absolute error:

$$c = c^{*} + \varepsilon_{abs},$$
$$\varepsilon_{abs} = \frac{4C.\Phi''}{c^{*} + \sqrt{c^{*2} + 8C.\Phi''}} \tag{2.3.8}$$

For the moment, to finish, just assume, see further the mass steady equation:

$$0 < \Phi'' = \frac{2G}{r.\sigma}\Phi < \frac{2G}{r.\sigma}\Phi^{A} \tag{2.3.9}$$

We can finally conclude that :

$$If\ \alpha \ll 1\ then\ c = Cte = c_{*} \tag{2.3.10}$$

Some numerical applications are necessary to obtain the order of magnitude of the quantities $c^{*},\ C\ and\ \alpha_{abs}$ (Table 2).

Thus, in both cases, it appears clearly that the error is absolutely negligible and this leads to a constant distribution of the concentrations along the gland's axis, remarkably almost equal, for chloride ions, to the concentration in the interstitium (bath): ~120 *mmol/L.* Table 2 illustrates this remarkable result and recalls the shape of the distribution of the concentration of chlorides in the healthy physiological state: isotonic filtration in the secretory coil and reabsorption in the excretory duct. Uniqueness of this solution is ensured since $C \ll 1$ and all the other terms in equation (2.3.6) of the results proof (i) are bounded and of the order of ~1. This simple result is noticeable and will simplify the mass equation because the equilibrium potential will be null.

2.4 Mass Steady Equation

For the proton, there is no dedicated channel; it is a "passe-partout". For chloride ions, the steady state can be expressed as $c(x) = Cte = c^{ext}$, which implies that the equilibrium (Nernst) potential, see part *1.2 The current*, is constant and almost null:

$$\Phi_{k} = Cte \approx 0. \tag{2.4.1}$$

So that in all cases, the conservation of mass is reduced to:

$$\frac{d(c.u)}{dx} = \frac{-2.z}{r.F}.G.\Phi \tag{2.4.2}$$

Taking into account Ohm's law $\left(c.u = \frac{\sigma}{F}.\frac{d\Phi}{dx}\right)$, at steady state, the potential can be depicted using an ordinary (non-linear) differential equation:

$$\frac{d^2\Phi}{dx^2} = \frac{2}{r.\sigma}.\ G.\Phi, \tag{2.4.3}$$

with $G = G(\Phi)$. Hence, a complete decoupling between the equations is observed and the whole problem can be solved sequentially: first, the momentum balance yields the constant concentration, then the mass equation (3.1.2) will lead to the potential, and finally the velocity can be deduced from Ohm's law.

3. Steady State additional analytics for the Conservation of Mass Equation

3.1 Continuity

Considering the steady conservation of mass equations just obtained, augmented with suitable boundary conditions, and recalling that no steady contact discontinuity in a medium with moving species, as seen in *result proof (ii)*, then the model reduces to the system of differential equations, with notations from Figure 1.

Current continuity at the entry of the duct:

$$\frac{d\Phi_e}{dx}(0) = \frac{\Phi_e(0)-\Phi^A}{h} \tag{3.1.1}$$

Balance in the duct:

$$\frac{d^2\Phi_e}{dx^2} = \frac{2}{\sigma.r}G_e\left(\Phi_e\right).\Phi_e \tag{3.1.2}$$

Combine with the coil (continuity of current and potential):

$$r_e^2 \frac{d\Phi_e}{dx}\left(L_e\right) = r_s^2 \frac{d\Phi_s}{dx}\left(L_e\right),\ \Phi_e\left(L_e\right) = \Phi_s\left(L_e\right) \quad (3.1.3)$$

Balance in the coil:

$$\frac{d^2\Phi_s}{dx^2} = \frac{2}{\sigma.r} G_s\left(\Phi_s\right).\Phi_s \quad (3.1.4)$$

Current continuity at the end of the coil:

$$\frac{d\Phi_s}{dx}\left(L_e + L_s\right) = -\frac{G_s\left(\Phi_s\left(L_e+L_s\right)\right).\Phi_s\left(L_e+L_s\right)}{\sigma} \quad (3.1.5)$$

To progress in the exploration for the last equations, a minor but quite obvious assumption is necessary:

$$\forall \Phi > 0,\ G(\Phi) > 0 \ and\ G\ increasing \quad (3.1.6)$$

Then the first lemma can be proven:

$$\forall x,\ \Phi(x) \leq \ \Phi(0) \leq \Phi^A \ and\ \Phi\ decreasing \quad (3.1.7)$$

To demonstrate the *result proof (ii)* we can write :

(3.1.8)

$\Phi_s'' \geq 0 \Rightarrow \Phi_s'$ increasing but $\Phi_s'\left(L_e + L_s\right) \leq 0 \Rightarrow \Phi_s' \leq 0 \Rightarrow \Phi_s$ decreasing current continuity and $\Phi_s' \leq 0 \Rightarrow \Phi_e'(L_e \leq 0 \Rightarrow \Phi_e' \leq 0 \Rightarrow \Phi_e\ decreasing$ as $\Phi_e'(0) \leq 0\ and\ current\ continuity \Rightarrow \Phi_e(0) \leq \Phi^A$.

3.2 The Voltage inside the Gland is Almost Constant

In fact, because the human skin is electrically weakly conductive, the model solution can be shown to be quite close to the linearized problem. In a first step, we begin by the lemma: Consider the simplified linearized model (with the same upper boundary condition):

$$\frac{d^2\Phi^{simple}}{dx^2} = \frac{2}{\sigma.r}G_s\left(\Phi^A\right).\Phi^A = Cte, \quad (3.2.1)$$
$$\frac{d\Phi^{simple}}{dx}\left(L_e + L_s\right) = -\frac{G_s\left(\Phi^A\right).\Phi^A}{\sigma},$$

Then

$$\forall x,\ \Phi^{simple}(x) \leq \Phi^A\ and\ \Phi^{simple}\ decreasing, \quad (3.2.2)$$
$$\forall x,\ \Phi(x) \geq \Phi^{simple}(x)$$

Now, the important corollary can be stated:

We have

$$\forall x,\ \Phi^A.(1 - \varepsilon) \leq \Phi(x) \leq \Phi^A, \quad (3.2.3)$$

with

$$G_e = G_e\left(\Phi^A\right)\ and\ G_s = G_s\left(\Phi^A\right), \quad (3.2.4)$$
$$\varepsilon = \frac{G_s}{\sigma.r_s.r_e^2}\left[r_s^3\left(h + L_e\right) + r_e^2.L_s^2 + r_s.L_s\left(r_e^2 + 2r_s\left[h + L_e\right]\right)\right] +$$
$$\frac{G_e}{\sigma}\left[L_e\left(2h + L_e\right)\right]$$

The order of magnitude is given in Figure 4 where the parameters of the gland are taken from (Sato et al., 1989; Chizmadzhev et al., 1998). No doubt, $\varepsilon < 8\ \% \ll 1$. To illustrate this, see the Figure 5 (A) for realistic simulations corresponding to different patient status.

The *result proof (iii)* of the lemma and corollary can be demonstrated. Let us put :

$$D = \Phi^{simple} - \Phi \text{ then} \quad (3.2.5)$$

$$\forall x,\ \Phi(x) \leq \Phi(0) \leq \Phi^A\ and\ \Phi\ decreasing$$

$$\frac{d^2D}{dx^2} = \frac{2}{\sigma.r}\left[G\left(\Phi^A\right).\Phi^A - G(\Phi).\Phi\right] \geq 0 \Rightarrow \frac{dD}{dx}\ increasing.$$

$$But\ \frac{dD}{dx}\left(L_e + L_s\right) = - \frac{\left[G\left(\Phi^A\right).\Phi^A - G(\Phi).\Phi\right]}{\sigma} \leq 0 \Rightarrow D\ decreasing.$$

$$However\ D(0) = h\frac{dD}{dx}(0) \leq 0 \Rightarrow \forall x,\ D(x) \leq 0 : \Phi^{simple}(x) \leq \Phi(x).$$

We can solve exactly the linearized problem, we find:

$$\Phi^{simple}(x) = \ = \{\frac{\Phi^A}{\sigma r_e^2}(G_e(x^2 - 2(h + x)L_e)r_e + \sigma r_e^2 - (h + x)G_s r_s(2L_s + r_s)) \quad (3.2.6)$$

$$if\ x \leq L_e\ \frac{\Phi^A}{\sigma r_e^2 r_s}(r_e(-G_e L_e(2h + L_e) + \sigma r_e)r_s + G_s((x - L_e)r_e^2(x - L_e - (h + L_e)r_s^2(2L_s + r_s))),\ otherwise \quad (3.2.6)$$

These formula show easily that:

$$\forall x,\ \Phi^{simple}(x) \leq \Phi^A\ and\ \Phi^{simple}\ decreasing. \quad (3.2.7)$$

$$We\ can\ deduce\ \forall x,\ \Phi(x) \geq \Phi^{simple}\left(L_e + L_s\right)\ because\ \Phi^{simple}\ decreasing.$$

It remains just to calculate $\Phi^{simple}\left(L_e + L_s\right)$ to get ε… Because here, simply

$$\varepsilon = \frac{\Phi^A - \Phi^{simple}\left(L_e + L_s\right)}{\Phi^A}$$

3.3 The Axial Current is Almost Piecewise Linear

The important corollary continues as follows:

Let $I^a\left(\Phi^A, x\right)$ be the axial current and $I^{a\_simple}\left(\Phi^A, x\right)$ be the simplified current, then

$$I^{a\_simple}\left(\Phi^A, x\right) = \pi.\Phi^A.\{2G_e\left(\Phi^A\right).(-x + L_e)r_e + G_s\left(\Phi^A\right).r_s(2L_s + r_s), \quad (3.3.1)$$

$$if\ x \le L_e G_s\left(\Phi^A\right).r_s(-\ 2x + 2L_e + 2L_s + r_s),$$

And if $\varepsilon < 1$:

$$\forall x,\ I^{a\_simple}\left(\Phi^A.[1-\varepsilon],\ x\right) \le I^a\left(\Phi^A,\ x\right) \le I^{a\_simple}\left(\Phi^A,\ x\right) \tag{3.3.2}$$

The associated results proof (iii) can be demonstrated using the Ohm's law see (6.3):

$$I^{a\_simple} = -\ \sigma.\pi.r^2.\frac{d\Phi^{simple}}{dx}, \tag{3.3.3}$$

and begin by calculation I^{a_simple} from formula (6.21), we obtain the desired result. Next, we continue with the Ohm's law:

$$I^a = -\ \sigma.\pi.r^2.\frac{d\Phi}{dx}, \tag{3.3.4}$$

and using the balance equation (6.9), we have

$$-\frac{dI^a}{dx} = 2\pi.r.G(\Phi).\Phi, \tag{3.3.5}$$

$$\Rightarrow I^a\left(\Phi^A,\ x\right) = I^a\left(\Phi^A,\ L_e + L_s\right) + 2\pi.r.\int_x^{L_e+L_s} G(\Phi).\Phi.dx,$$

or

$$I^a\left(\Phi^A,\ L_e + L_s\right) = \pi.r^2.G\left(\Phi\left(L_e + L_s\right)\right).\Phi\left(L_e + L_s\right), \tag{3.3.6}$$

and

$$G\left(\Phi^A.[1-\varepsilon]\right).\Phi^A.[1-\varepsilon] \le G(\Phi).\Phi \le G\left(\Phi^A\right).\Phi^A \tag{3.3.7}$$

Figure 5 (B) confirms this result with realistic numerical simulations.

4. Skin Conductance: Gland Wall Ion Permeability

One parameter of the measured signal, among others, see Section *ESC parameters*, is the Electrochemical Skin Conductance (ESC). It is deduced from the (axial) current crossing the electrode and the applied voltage Φ^A. Because, from the previous formula :

$$I^{a\_simple}\left(\Phi^A, 0\right) = \pi.\left[2G_e\left(\Phi^A\right).L_e.r_e + G_s\left(\Phi^A\right).r_s(2L_s + r_s)\right].\Phi^A \quad (4.1)$$

The main theorem can be proven, notations by definition:

$$ESC\left(\Phi^A\right) = \frac{I^a\left(\Phi^A, 0\right)}{\Phi^A}, \quad (4.2)$$

The (total) conductance of the gland wall:

$$GWC\left(\Phi^A\right) = 2\pi.\ r_e.L_e.G_e\left(\Phi^A\right) + 2\pi.\ r_s.L_s.G_s\left(\Phi^A\right) + \pi.\ r_s^{\ 2}.G_s\left(\Phi^A\right) \quad (4.3)$$

Result:

$$(1 - \varepsilon)GWC\left(\Phi^A.[1 - \varepsilon]\right) \leq ESC(\Phi^A) \leq GWC\left(\Phi^A\right) \quad (4.4)$$

To demonstrate this, the previous result with the axial current can be used:

$$\forall x,\ I^{a\_simple}\left(\Phi^A.[1 - \varepsilon],\ x\right) \leq I^a\left(\Phi^A,\ x\right) \leq I^{a\_simple}\left(\Phi^A,\ x\right), \quad (4.5)$$
$$\Rightarrow I^{a\_simple}\left(\Phi^A.[1 - \varepsilon],\ 0\right) \leq I^a\left(\Phi^A,\ 0\right) \leq I^{a\_simple}\left(\Phi^A,\ 0\right),$$

and

$$I^{a\_simple}\left(\Phi^A, 0\right) = \pi.\left[2G_e\left(\Phi^A\right).L_e.r_e + G_s\left(\Phi^A\right).r_s(2L_s + r_s)\right].\Phi^A \quad (4.6)$$

5. Normalization

To conclude this demonstration, normalization of the conductances is necessary to refine results. The range of skin conductance is from a few µS to several hundred µS. The aim of this normalization is to pack very high and healthy conductances as well as stretch weak and problematic conductances. For a gross conductance Y expressed in µS, the normalization is defined by the increasing dimensionless bijection from $[0, \ +\infty[$ to $[0, \ 100[$:

$$Y_{norm} = \frac{100.Y}{X_{ref}+Y}, \tag{5.1}$$

where $X_{ref} = 40\ \mu S$. The theorem is written:

Given:

$$\theta = \frac{\varepsilon.X_{ref}}{X_{ref}+(1-\varepsilon).GWC\left(\Phi^{A}.[1-\varepsilon]\right)}, \tag{5.2}$$

where $GWC\left(\Phi^{A}\right)$ in μS. Then:

$$\forall\Phi^{A},\ (1-\theta).GWC_{norm}\left(\Phi^{A}.[1-\varepsilon]\right) \leq ESC_{norm}(\Phi^{A}) \leq\leq GWC_{norm}\left(\Phi^{A}\right) \tag{5.3}$$

The order of magnitude is given in Table 1 and, $\theta < 0.8\ \% < \varepsilon < 8\ \% \ll 1$. The second inequality is obvious thanks to the increasing bijection. For the first one, we have:

$$ESC_{norm}\left(\Phi^{A}\right) = \frac{100.ESC\left(\Phi^{A}\right)}{X_{ref}+ESC\left(\Phi^{A}\right)} \geq \frac{100.(1-\varepsilon)GWC\left(\Phi^{A}.[1-\varepsilon]\right)}{X_{ref}+(1-\varepsilon)GWC\left(\Phi^{A}.[1-\varepsilon]\right)} = \tag{5.4}$$
$$GWC_{norm}\left(\Phi^{A}.[1-\varepsilon]\right).\frac{(1-\varepsilon)(X_{ref}+GWC\left(\Phi^{A}.[1-\varepsilon]\right))}{X_{ref}+(1-\varepsilon)GWC\left(\Phi^{A}.[1-\varepsilon]\right)} =$$
$$(1-\theta).GWC_{norm}\left(\Phi^{A}.[1-\varepsilon]\right)$$

For a result example see ESC parameters - main parameters section within the publication.

Others parameters

The offset, Oa: results from the overpotential (voltage consumption, diode effect) omnipresent at the anode in electrochemistry, due to the electrode surface oxidation and corresponding to the bottom of the oxidation wall. It depends in particular, see *Virgine Lair et al.* (Lair et al., 2019), on certain concentrations of the electrolytes in sweat (chlorides Cl^- and carbonates HCO_3^-) and the material composition of the electrode. It can also be affected by certain pathologies or treatments. The first voltage step is not used directly, but it stabilizes the overpotential and offset due to its extended duration of eight seconds instead of the usual one second. The last level is also not used; instead, it confirms that the measured signal has not reached the oxidative wall. Once the offset has stabilized, the entire measured signal is just a simple shift, without distortion, of the purely physiological signal. On the other hand, as the lower part of the signal is generally – and is assumed and found to be – linear, this offset is obtained by extending the slope of the low conductance towards the abscissa axis, by (Figure 5) :

$$Oa = \phi_{14}^{A} - \phi_{14}^{X} - \frac{I_{14}}{LC}$$

By shifting the signal of the offset, the conductance C_s for any step s is then deduced simply by the chord:

$$C_s = \frac{I_s}{\phi_s^A - \phi_s^X}$$

The high conductance HC: is defined by:

$$HC = C_2$$

The detachment ratio r:

$$r = \frac{HC}{LC}$$

The coefficient of non-linearity ρ: quantifying the non-linearity at the bottom of the curve (~ second derivative):

$$\rho = \frac{\Delta C12, 13}{\Delta C13, 14}$$

So if $\rho \approx 1$: linear lower part, if $\rho > 1$: early detachment case and if $\rho < 1$: case of early landing.

The hand to foot ratio R:

$$R = \frac{LC_{hand}}{LC_{foot}}$$

At the cathode, the formulas are analogous. Note that the offset on the cathode side is very low because the first step allows the regeneration (Ayoub et al., 2012) of this electrode.

The main parameter now has an extensive bibliography (see State of the art) but the others have currently no validation. Researches are conducted to evaluate their performance into specific conditions but for now they cannot be used or only for validation or early research

Figures

Figure 1

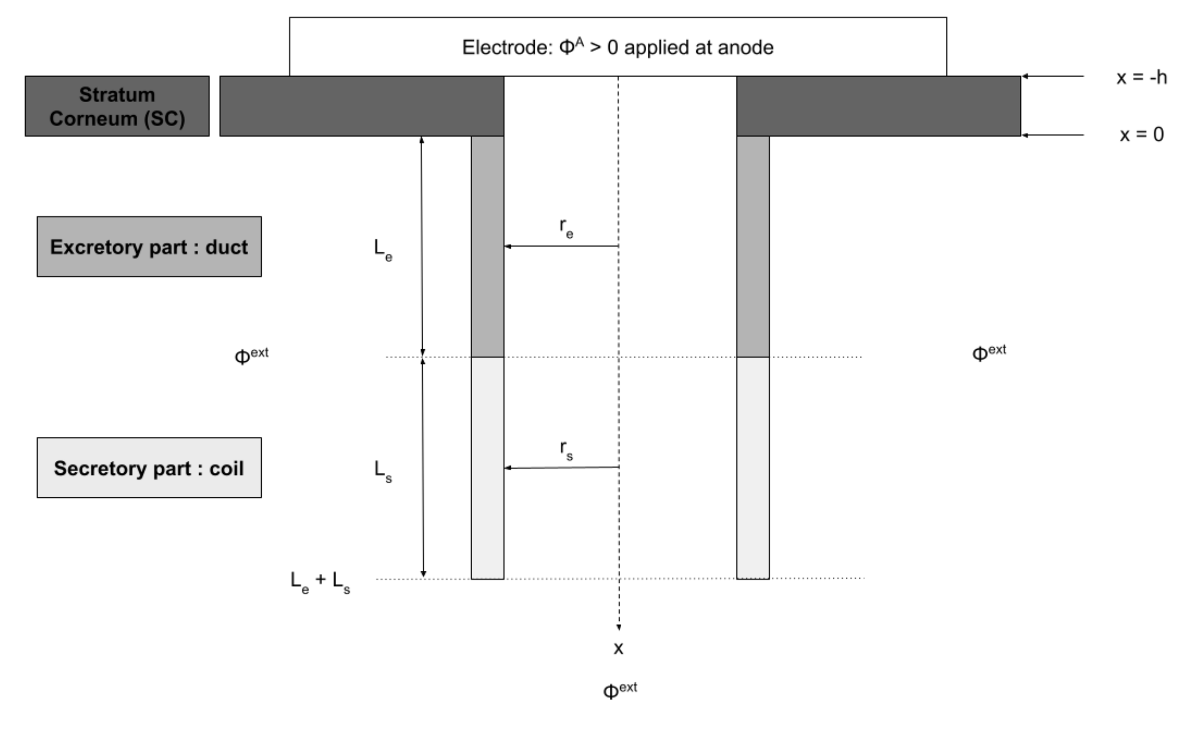

Figure 1. Geometrical model of the sweat gland.

Figure 2

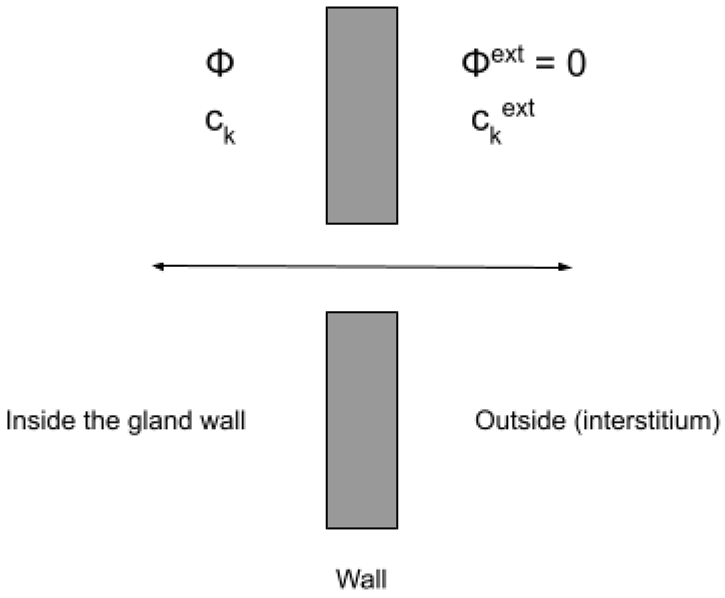

Figure 2. Channel across the gland wall.

Figure 3

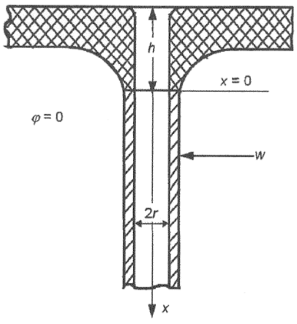

experimental protocol. The potential distribution along x can be found from the balance equation for electric current averaged over the tube cross section

$$C_w \frac{\partial \varphi}{\partial t} = \frac{\sigma r}{2} \frac{\partial^2 \varphi}{\partial x^2} - G_w \varphi \qquad (8)$$

with initial and boundary conditions

$$\varphi|_{t=0} = 0, \quad \varphi|_{x\to\infty} = 0, \quad \text{and} \quad \left(\varphi - h\frac{\partial \varphi}{\partial x}\right)\Big|_{x=0} = U \qquad (9)$$

The last condition is a consequence of zero wall conductance in the upper, SC-lined region ($-h \le x \le 0$). We neglect the potential drop across the resistance $R_b + R_c$,

Figure 3. Extract from Chizmazdhev

Figure 4

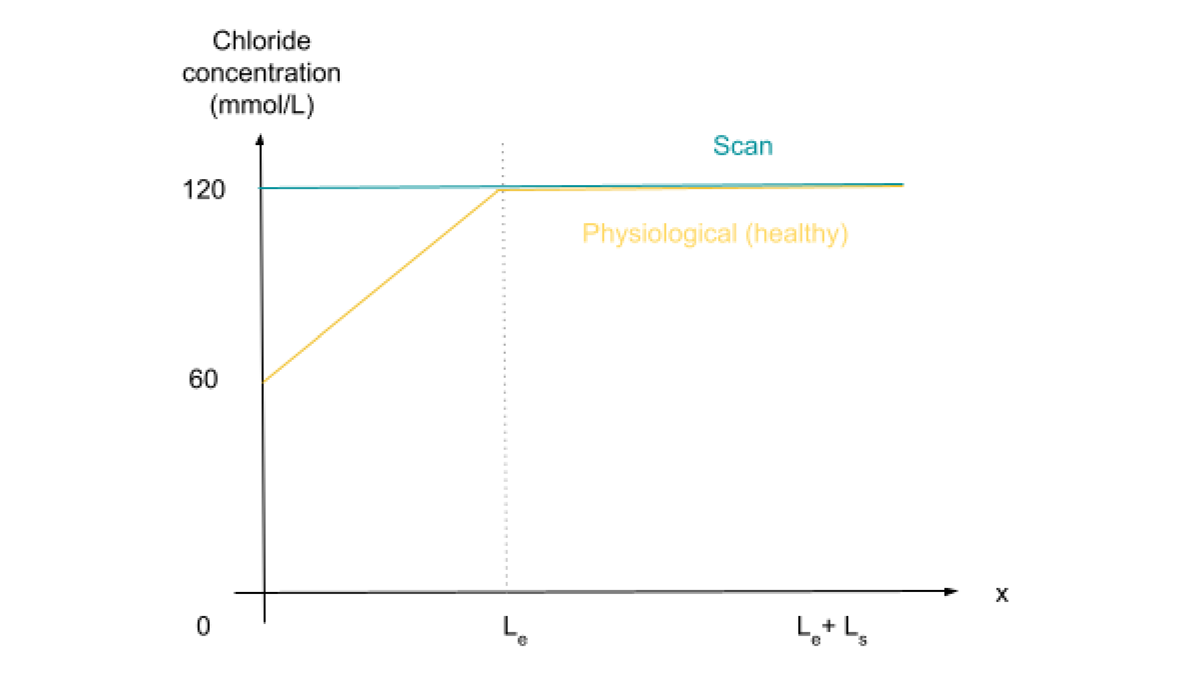

Figure 4. Chloride concentration depending on L parameters.

Figure 5

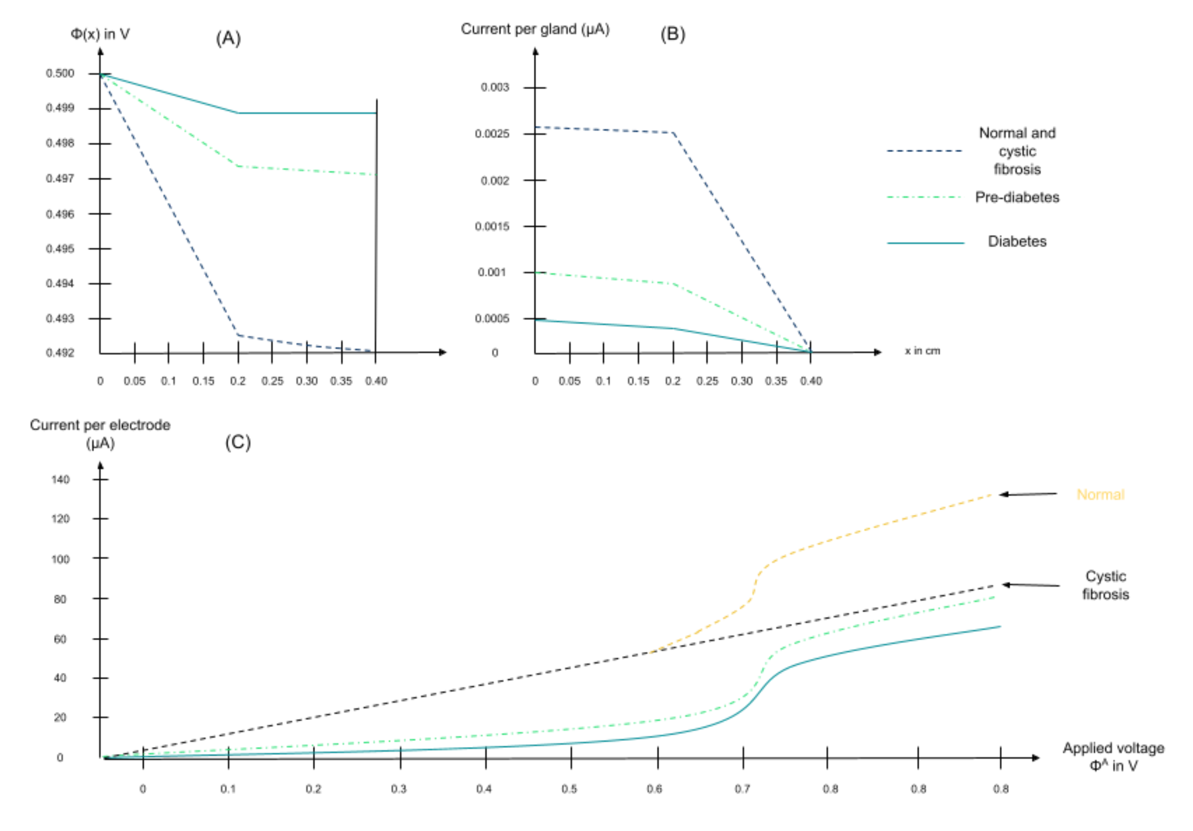

Figure 5. Realistic numerical simulations.

Tables

Table 1

Table 1. Numerical application for Cl- near the anode and H+near the cathode.

**General data**			**Ions data input**		
μ [kg/(m.s)]	1,0000E-03			**Cl-**	**H+**
σ [S/cm]	1,0000E-02		***M [m2/(s.V)***	7,9800E-08	3,6230E-07
e [C]	1,6022E-19		***H [m]***	1,0000E-10	2,3459E-11
F [C/mol]	9,6485E+04		***M [Kg/mol]***	3,5000E-02	1,0000E-03
N	6,0224E+23				
r [cm]	1,0000E-03		**Ion data output**		
Gmax [μS/cm2]	1,0000E+01			**Cl-**	**H+**
Φmax [V]	1,0000E+00		***m [kg]***	5,8116E-26	1,6605E-27
			ζ	2,0077E-12	4,4222E-13
			C	3,8964E-17	1,1133E-18
			c* [mmol/L]	1,2988E+02	2,6610E+01
			α_(abs) [mmol/L]	1,4211E-14	-1,7764E-15
			α_(relat) [%]	1,0942E-14	-6,2095E-15

Bibliography

Atkins, P. W., and Paula, J. D. (2017). *Elements of Physical Chemistry*. Oxford University Press.

Ayoub, H., Calvet, J. H., Lair, V., Griveau, S., Bedioui, F., Cassir, M., et al. (2012). "Electrochemical Basis for EZSCAN/SUDOSCAN: A Quick, Simple, and Non-Invasive Method to Evaluate Sudomotor Dysfunctions," in *Developments in Electrochemistry*, (IntechOpen). doi: 10.5772/53965

Chen, T. Y., and Miller, C. (1996). Nonequilibrium gating and voltage dependence of the ClC-0 Cl- channel. *J. Gen. Physiol.* 108, 237–250. doi: 10.1085/jgp.108.4.237

Chizmadzhev, Y. A., Indenbom, A. V., Kuzmin, P. I., Galichenko, S. V., Weaver, J. C., and Potts, R. O. (1998). Electrical properties of skin at moderate voltages: contribution of appendageal macropores. *Biophys. J.* 74, 843–856. doi: 10.1016/S0006-3495(98)74008-1

Cronin, J. (1981). *Mathematics of Cell Electrophysiology*. CRC Press.

Granger, D., Marsolais, M., Burry, J., and Laprade, R. (2003). Na+/H+ exchangers in the human eccrine sweat duct. *Am. J. Physiol. Cell Physiol.* 285, C1047-1058. doi: 10.1152/ajpcell.00581.2002

Lair, V., Calmet, A., Albin, V., Griveau, S., and Cassir, M. (2019). Electrolytic Cell Design to Simulate the Electrochemical Skin Response. *Electroanalysis* 31, 22–30. doi: 10.1002/elan.201800504

Layton, E. M. Jr. (1975). Modern Electrochemistry (Bockris, John O'M.; Reddy, Amulya K. N.). *J. Chem. Educ.* 52, A60. doi: 10.1021/ed052pA60.2

Sato, K., Kang, W. H., Saga, K., and Sato, K. T. (1989). Biology of sweat glands and their disorders. I. Normal sweat gland function. *J. Am. Acad. Dermatol.* 20, 537–563. doi: 10.1016/s0190-9622(89)70063-3

Wills, N. K., and Fong, P. (2001). ClC chloride channels in epithelia: recent progress and remaining puzzles. *News Physiol. Sci. Int. J. Physiol. Prod. Jointly Int. Union Physiol. Sci. Am. Physiol. Soc.* 16, 161–166. doi: 10.1152/physiologyonline.2001.16.4.161
